# Comparison of ^18^F-Choline PET/CT and ^99m^Tc-Sestamibi SPECT/CT in the Localization of Hyperactive Parathyroid Glands in Primary Hyperparathyroidism: Diagnostic Performance in Discordant Cases

**DOI:** 10.3389/fendo.2025.1677278

**Published:** 2025-11-13

**Authors:** Sungkeun Kang, Hojin Cho, Eunjin Kim, Jin Kyong Kim, Sang-wook Kang, Jong Ju Jeong, Kee-Hyun Nam, Woungyoun Chung

**Affiliations:** 1 Division of Thyroid-Endocrine Surgery, Department of Surgery, Severance Hospital, Yonsei University College of Medicine, Seoul, Republic of Korea; 2 Department of Nuclear Medicine, Severance Hospital, Yonsei University College of Medicine, Seoul, Republic of Korea

**Keywords:** Hyperparathyroidism, Parathyroidectomy, ^99m^Tc sestamibi SPECT/CT, ^18^-F-fluorocholine PET/CT, localization

## Abstract

**Introduction:**

Accurate localization of hyperactive parathyroid glands is essential in managing primary hyperparathyroidism. Despite advances in imaging, discordant findings still complicate surgical planning. Tc-99m-sestaMIBI SPECT/CT (MIBI SPECT/CT) and ^18^F-Choline PET/CT (FCH PET/CT) are commonly used, particularly when localization is discordant.

**Objective:**

This study compared the findings of FCH PET/CT and MIBI SPECT/CT with intraoperative outcomes in patients who underwent parathyroidectomy for primary hyperparathyroidism. In discordant cases, the relative diagnostic performance and postoperative outcomes of the two modalities were analyzed.

**Materials and methods:**

We retrospectively reviewed 133 patients who underwent parathyroidectomy between January 2020 and December 2024 and had both MIBI SPECT/CT and FCH PET/CT. Patients were classified according to concordance between imaging and surgical localization: Group 1 (both modalities concordant), Group 2 (MIBI concordant only), Group 3 (FCH concordant only), and Group 4 (both discordant). Diagnostic performance (sensitivity and PPV) and biochemical cure—defined as normalization of parathyroid hormone (PTH) and calcium at 6 and 12 months—were compared between Groups 2 and 3.

**Results:**

Of 133 patients, 82 (61.7%) were in Group 1, 5 (3.8%) in Group 2, 37 (27.8%) in Group 3, and 9 (6.8%) in Group 4. Sensitivity and PPV were 74.4% and 86.1% for MIBI, and 97.5% and 91.5% for FCH PET/CT, respectively. The biochemical cure rate was 80.0% vs 94.6% at 6 months and 50.0% vs 87.5% at 12 months for Groups 2 and 3, with calcium levels remaining within the normal range. Preoperative PTH levels and chief-cell proportions were higher in Group 3 (91.9%) than in Group 1 (82.6%).

**Discussion:**

FCH PET/CT demonstrated better diagnostic performance and higher cure rates than MIBI SPECT/CT in discordant cases. Although differences were not statistically significant, they may be influenced by preoperative parathyroid activity and histologic composition.

**Conclusion:**

FCH PET/CT showed a trend toward improved diagnostic performance and postoperative cure compared with MIBI SPECT/CT in discordant cases. While not superior in all situations, it can complement MIBI SPECT/CT and enhance surgical decision-making in complex clinical settings.

## Introduction

Primary hyperparathyroidism (PHPT) is a common endocrine disorder characterized by excessive secretion of parathyroid hormone (PTH), most frequently due to a solitary adenoma (1, 2). Untreated PHPT can lead to serious clinical complications, ranging from osteoporosis and renal stones to neuromuscular symptoms (2, 3). Surgical resection of the hyperfunctioning gland remains the definitive treatment (4), and precise preoperative localization is essential for the success of minimally invasive parathyroidectomy (1, 3, 5).

The biological behavior of hyperfunctioning parathyroid glands is closely related to their cellular composition, particularly the relative proportions of chief and oxyphilic cells. These histologic characteristics influence both tracer uptake and diagnostic accuracy (6).

Nevertheless, accurate localization can remain difficult, particularly in cases with multi-gland disease, ectopic gland location, or when imaging results are equivocal or inconclusive (7). Due to these challenging situations, multiple localization strategies have been implemented (7, 8), including neck ultrasonography, 99mTc-sestamibi single-photon emission computed tomography/computed tomography (MIBI SPECT/CT), 18F-fluorocholine positron emission tomography/computed tomography (FCH PET/CT), four-dimensional computed tomography (4D CT), parathyroid venous sampling (PVS), fine needle aspiration biopsy (FNAB), intraoperative PTH monitoring, and frozen section biopsy. Despite this multimodal approach, discordant imaging findings remain common in clinical practice and often complicate surgical decision-making (9–11).

Consequently, determining the most accurate imaging modality in discordant cases is a critical consideration. Although both MIBI SPECT/CT and FCH PET/CT are widely used, their comparative diagnostic performance in such scenarios remains unclear (9, 12). Discordant cases are clinically challenging because they complicate surgical planning, and prior studies and meta-analyses have rarely examined this subgroup. Our study addresses this gap by examining only discordant imaging cases, with the goal of adding evidence that builds on recent multicenter and meta-analytic work and drawing attention to the particular challenges of this underrepresented group. We also sought to evaluate the diagnostic accuracy and clinical outcomes of each modality and to explore factors that may influence their performance.

## Materials and methods

### Study design and population

**Figure 1:**
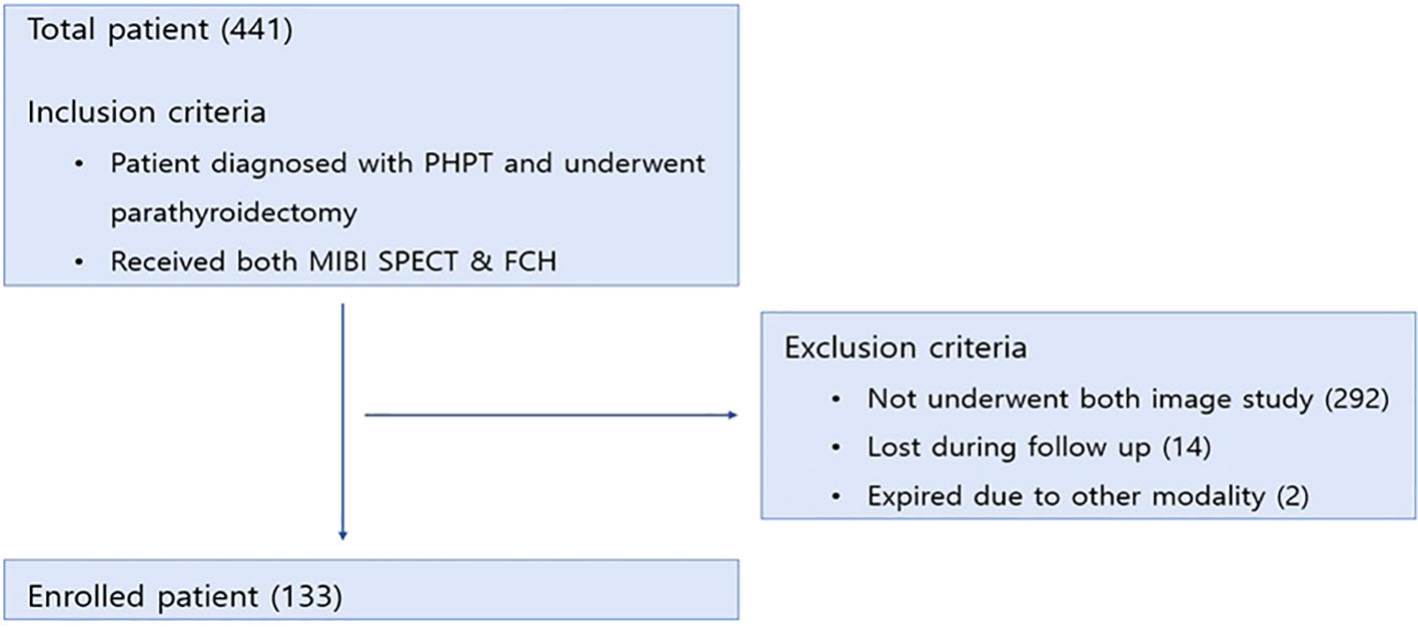
Flow diagram of patient enrollment and exclusion.

This retrospective study was conducted at Severance Hospital, Yonsei University College of Medicine. Medical records of patients who underwent parathyroidectomy for primary hyperparathyroidism between January 2020 and December 2024 were reviewed. Among the initial 441 candidates, 133 patients were ultimately enrolled (**Figure 1**). Eligible patients were those diagnosed with primary hyperparathyroidism who subsequently underwent parathyroidectomy and received preoperative evaluation with both MIBI SPECT/CT and FCH PET/CT. Patients were excluded if they had not undergone both imaging modalities (n = 292), were lost to follow-up after surgery (n = 14), or died due to unrelated comorbidities (n = 2).

### Image and surgical protocol

All patients underwent preoperative imaging using both MIBI SPECT/CT and FCH PET/CT. For MIBI imaging, each patient received an intravenous injection of 740 ± 20 MBq of 99mTc-MIBI. Early and delayed planar images were acquired at 10 minutes and 2 hours post-injection, respectively. Planar images were obtained in anterior, 20° right anterior oblique, and 20° left anterior oblique views using a Symbia TruePoint SPECT/CT system (Siemens Healthcare, Knoxville, TN, USA). Immediately after delayed planar imaging, SPECT and CT scans were performed. CT scans were acquired with a slice thickness of 5 mm, tube voltage of 110 kV, and tube current of 42 mA, using a 512 × 512 matrix and a standard reconstruction filter. SPECT imaging of the neck was performed with a zoom factor of 1.78 and a 128 × 128 matrix using a step-and-shoot acquisition mode (32 frames, 25 seconds per frame, 5.5° angular step). SPECT image reconstruction included CT-based attenuation correction and a three-dimensional Hanning post-filter (cutoff frequency: 0.85 cycles/cm), with 3D Flash iterative reconstruction (8 subsets, 8 iterations). The SPECT acquisition took approximately 14 minutes, while the CT acquisition required 10–20 seconds. For FCH PET/CT imaging, all patients fasted for at least 6 hours prior to the scan. Each patient received an intravenous injection of 18F-fluorocholine at a dose of 3.0 MBq/kg. After a 1-hour uptake period, PET/CT scans were performed using commercial PET/CT scanners (Discovery D600 or Discovery D710, GE Healthcare, Chicago, IL, USA). PET acquisition was conducted in 3D mode with an acquisition time of 2 minutes per bed position. PET images were reconstructed using an ordered subset expectation maximization (OSEM) algorithm, with attenuation correction based on the low-dose CT dataset. Image interpretation for both modalities was conducted by board-certified nuclear medicine physicians who were blinded to surgical outcomes. A positive localization was defined as a focal uptake greater than background thyroid tissue at a site consistent with a parathyroid gland. For MIBI SPECT/CT, uptake had to persist on delayed images and be confirmed on fused SPECT/CT. For FCH PET/CT, positivity was defined as a discrete focus of increased choline uptake not explained by physiological structures. Any discrepancies were resolved by consensus between readers. Interval between imaging study and operation is less than 3 months.

All patients subsequently underwent open selective parathyroidectomy performed by experienced endocrine surgeons. When preoperative imaging provided clear localization, patients underwent focused parathyroidectomy. In cases where localization was uncertain or difficult, a larger incision with unilateral or bilateral neck exploration was performed according to the location of the suspected lesions. Localization during surgery was primarily guided by preoperative imaging findings. Neck ultrasonography and intraoperative PTH monitoring were performed in all patients and contributed to preoperative localization planning. As needed, adjunctive localization methods—such as 4D CT, PVS, and frozen section biopsy—were additionally employed to assist in identifying hyperfunctioning glands. Final intraoperative findings and histopathologic results served as the reference standard for evaluating the diagnostic performance of each imaging modality.

### Group classification

In this study, concordance between imaging findings and surgical outcomes was assessed by comparing the anatomical localization of the hyperfunctioning parathyroid gland. Concordance was defined when both preoperative imaging and intraoperative findings matched in laterality (left vs. right) and vertical position (upper vs. lower), as confirmed by surgical exploration and pathological examination. For consistency, imaging findings were reviewed with nuclear medicine physicians and recorded using a four-quadrant system (left/right, upper/lower). A case was classified as concordant when imaging and surgical/pathological findings matched within the same quadrant(s). Any mismatch in quadrant assignment was defined as discordant. In addition, when imaging and surgical findings both suggested multiple abnormal glands but the quadrants did not match exactly, these cases were also defined as discordant (e.g., uptake in the left superior quadrant on FCH PET/CT, but both left superior and left inferior glands identified as pathological at surgery).

Based on the concordance status of MIBI SPECT/CT and FCH PET/CT, patients were categorized into four groups:

- Group 1: Both imaging modalities were concordant.
- Group 2: Only MIBI SPECT/CT was concordant.
- Group 3: Only FCH PET/CT was concordant.
- Group 4: Both modalities were discordant.

### Diagnostic performance and cure rate

This study evaluated the diagnostic performance and cure rates of MIBI SPECT/CT and FCH PET/CT in discordant cases. Diagnostic performance was assessed by calculating sensitivity and positive predictive value (PPV) for each imaging modality. Biochemical cure was defined as normalization of serum PTH (≤65 pg/mL) and calcium levels within the normal range (8.5mg/dL-10.5mg/dL). Serum calcium was measured preoperatively, on the day of surgery, and at postoperative 6-month and 12-month follow-up visits. Outcomes were assessed at both short-term (6 months) and long-term (12 months) follow-up. PTH levels were measured at multiple time points. Preoperative PTH was assessed during initial laboratory evaluation. Intraoperative PTH was measured at 5 and 15 minutes after excision of the suspected pathologic parathyroid gland. Intraoperative biochemical success was defined as a ≥50% decline from the highest pre-excision value at 10–15 minutes (Miami criterion) and/or a decline into the normal range, and was used to guide surgical decision-making. Postoperative PTH was measured 6 months and 12 months after surgery to assess biochemical outcome. Pathological diagnosis was confirmed by a board-certified pathologist based on postoperative tissue specimens. In addition, laboratory findings and pathological characteristics were analyzed to identify potential factors affecting diagnostic performance between groups.

### Statistical analysis

Sensitivity and PPV of each imaging modality were calculated based on intraoperative findings and final pathological confirmation, and corresponding 95% confidence intervals (CIs) were derived using the Clopper–Pearson exact method. Because our study cohort included only patients with surgically and pathologically confirmed primary hyperparathyroidism, true-negative cases were not available. As a result, specificity and negative predictive value (NPV) could not be calculated, and our analysis was therefore limited to sensitivity and PPV. Categorical variables were compared using the chi-square test or Fisher’s exact test, and continuous variables were analyzed using the Student’s t-test or Mann–Whitney U test, as appropriate. In subgroup analyses, cell composition (chief vs. oxyphilic cell proportions) between groups was compared using the Mann–Whitney U test, and pathologic types (adenoma vs. hyperplasia) were compared using Fisher’s exact test. A p-value less than 0.05 was considered statistically significant.

## Results

A total of 133 patients were included in the analysis. The mean age was 57.66 (range, 14–86 years), and the majority were female (77.44%). The mean BMI was 24.64kg/m² (range, 16.34–40.93). Most patients (99.25%) had no prior history of neck surgery. Preoperative median serum PTH level was 149.93 pg/mL (range, 58.40–966.00). Intraoperative PTH levels decreased to a median of 54.70 pg/mL at 5 minutes and 31.50 pg/mL at 15 minutes post-excision. At 6 months postoperatively, the median PTH level was 47.47 pg/mL and 45.20 pg/mL at 12 months. Serum calcium levels decreased to a median of 9.99 mg/dL immediately after surgery. At 6 months and 12 months postoperatively, the median calcium levels were 9.37 mg/dL and 9.37 mg/dL, respectively, remaining within the normal range. Final pathology revealed parathyroid adenoma in 89.47% of patients, hyperplasia in 10.53%. With respect to the number of operated glands, 127 patients (95.49%) underwent single-gland resection, whereas 6 patients (4.51%) required double-gland resection. (**Table 1**).

**Table 1:** Patient characteristics.

	N	%	Median	SD(±)	Minimal	Maximal
Total patients	133					
Age			57.56	14.37	14	86
Gender						
Male	30	22.56				
Female	103	77.44				
BMI			24.64	4.53	16.34	40.93
Neck OP history						
None	132	99.25				
More than once	1	0.75				
PTH level (ng/ml)						
Pre-op			149.93	119.38	58.40	966.00
Intra-op						
5 min			54.70	39.43	11.10	184.00
15 min			31.50	25.39	5.50	205.00
Post op						
6 months			47.47	24.49	15.60	152.00
12 months			45.20	23.12	20.30	113.00
Calcium level (mg/dL)						
Pre-op			11.03	0.69	9.00	12.90
Post op						
Post op			9.99	0.86	8.40	11.80
6 months			9.37	0.41	8.60	10.50
12 months			9.36	0.40	8.40	10.30
Operated Glands						
Single gland	127	95.49				
Double glands	6	4.51				
Pathology						
Adenoma	119	89.47				
Hyperplasia	14	10.53				

Categorical variables are shown as number (%). Continuous variables are presented as median, standard deviation (SD), minimum, and maximum. PTH levels were measured preoperatively, intraoperatively (5 and 15 minutes), and postoperatively at 6 and 12 months. Calcium levels were measured preoperatively, immediately after surgery, and at 6 and 12 months.

Among the 133 patients, four groups were classified according to the concordance between preoperative imaging findings and intraoperative localization. Group 1 included 82 patients (61.65%) in whom both MIBI SPECT/CT and FCH PET/CT were concordant with surgical findings. Group 2 consisted of 5 patients (3.76%) showing concordance only with MIBI SPECT/CT, while Group 3 included 37 patients (27.82%) in whom only FCH PET/CT matched the operative findings. Group 4 comprised 9 patients (6.77%) in whom both imaging modalities were discordant with the intraoperative localization (**Table 2**).

**Table 2:** Grouping based on the consistency between preooperative MIBI SPECT/CT & FCH PET/CT results, and operational result.

Group	OP-SPECT result	OP-Choline PET result	Number	(%)
1	Concordance	Concordance	82	61.65%
2	Concordance	Discordance	5	3.76%
3	Discordance	Concordance	37	27.82%
4	Discordance	Discordance	9	6.77%

This table provides descriptive information only; no statistical comparisons were performed between groups.

Concordance was defined as agreement in quadrant location (left/right, superior/inferior).

In discordant cases, FCH PET/CT demonstrated superior diagnostic performance compared to MIBI SPECT/CT. The sensitivity of FCH PET/CT was 97.54% (95% CI, 93.0–99.5%), and its PPV was 91.54% (95% CI, 85.4–95.7%). In contrast, MIBI SPECT/CT showed a sensitivity of 74.36% (95% CI, 65.5–81.9%) and a PPV of 86.14% (95% CI, 77.8–92.2%) (**Table 3**).

**Table 3:** Diagnostic performance of MIBI SPECT/CT & FCH PET/CT.

	Sensitivity % (95% CI)	Specificty %	PPV % (95% CI)	NPV %
MIBI SPECT	74.36 (65.5–81.9)	NA (0%)	86.14 (77.8–92.2)	NA (0%)
FCH	97.54 (93.0–99.5)	NA (0%)	91.54 (85.4–95.7)	NA (0%)

95% CI calculated using Clopper–Pearson method.

NA, Not applicable.

Because the study cohort included only surgically and pathologically confirmed cases (no true-negative subjects), specificity and NPV were not estimable (TN = 0).

Neck ultrasonography was performed in all patients. Concordance with surgical localization was observed in 71 of 133 patients (53.4%), while 62 patients (46.6%) showed discordance.

Postoperative biochemical cure was assessed using both serum PTH and calcium levels at short-term (6 months) and long-term (12 months) follow-up. In Group 2, the cure rate was 80.0% at 6 months, while in Group 3, the cure rate was 94.59%. At 12 months, PTH normalization was observed in 50.0% of Group 2 patients and 87.5% of Group 3 patients. Calcium demonstrated excellent control across both groups: 97.5% vs. 100% at 6 months and 100% vs. 100% at 12 months for Groups 2 and 3, respectively. At 12 months, six patients demonstrated elevated PTH despite normal calcium levels, all of whom were found to have vitamin D deficiency, indicating that the biochemical findings were attributable to secondary causes. Perioperative changes in PTH levels were compared between Group 2 (MIBI concordant only) and Group 3 (FCH PET/CT concordant only). Group 3 showed higher preoperative PTH levels (134.1 pg/mL) compared to Group 2 (114.2 pg/mL). In both groups, PTH levels decreased markedly after gland excision, reaching their lowest point at 15 minutes intraoperatively (Group 3: 30.5 pg/mL; Group 2: 27.5 pg/mL), and subsequently rose at 6 months postoperatively (Group 3: 42.2 pg/mL; Group 2: 51.5 pg/mL). This mild postoperative increase remained within the normal range and was not considered clinically significant. The overall trend showed that patients with higher preoperative PTH levels—predominantly seen in Group 3—demonstrated better concordance with FCH PET/CT than with MIBI SPECT/CT.

To evaluate potential pathologic factors affecting diagnostic accuracy, we compared histologic findings between Group 1 (concordant in both MIBI SPECT/CT and FCH PET/CT) and Group 3 (concordant only in FCH PET/CT). Group 1 was chosen as the reference group instead of Group 2, as it represents a fully concordant population without discordance-related bias. Compared to Group 1, Group 3 demonstrated a higher proportion of chief cells (91.94% vs. 82.65%, p-value: 0.098) and a lower proportion of oxyphilic cells (9.03% vs. 18.26%). Adenoma was the most common diagnosis in both groups, with 74 cases in Group 1 and 33 in Group 3. Hyperplasia was observed in 8 and 4 patients, respectively (**Table 4**).

**Table 4:** Pathologic cell composition ratio and types in group 2 & group 3.

	Group 1	Group 3	P-Value
Cell composition			0.098
Chief (%)	82.65	91.94	
Oxiphil (%)	18.26	9.03	
Pathlogic types (n)			1.000
Adenoma	74	33	
Hyperplasia	8	4	

p-values calculated by Mann–Whitney U test for continuous variables and Fisher’s exact test for categorical variables.

## Discussion

In this study, we compared the diagnostic performance and clinical outcomes of FCH PET/CT and MIBI SPECT/CT in patients with primary hyperparathyroidism, with a focus on cases with discordant localization. Our findings demonstrated that diagnostic performance and cure rate were superior in FCH PET/CT than MIBI SPECT/CT. Specifically, FCH PET/CT showed a sensitivity of 97.54% and a PPV of 91.54%, compared to 74.36% and 86.14%, respectively, for MIBI SPECT/CT. Additionally, patients in the FCH PET/CT-concordant group (Group 3) achieved higher biochemical cure rates than those in the MIBI-concordant group (Group 2). At 6 months, PTH normalization was observed in 94.6% of Group 3 and 80.0% of Group 2 patients. At 12 months, the PTH cure rates declined to 87.5% and 50.0%, respectively. These findings indicate only a trend favoring Group 3; however, given the very small number of patients in Group 2 (n=5), these comparisons should be interpreted with caution. Calcium levels remained excellent in both groups: 100% in Group 3 and 97.5% in Group 2 at 6 months, and 100% in both groups at 12 months. Six patients showed elevated PTH despite normal calcium at 12 months, and all were found to have vitamin D deficiency, indicating that the abnormal PTH values were likely related to secondary factors rather than recurrent disease.

Intraoperative PTH is a widely used surrogate of surgical success but cannot fully exclude late recurrence. Misclassification is possible, as elevated PTH with normal calcium may reflect secondary causes such as vitamin D deficiency. To reduce this risk, we reported both PTH and calcium outcomes at 6 and 12 months, and noted that the six normocalcemic PTH elevations at 12 months were due to vitamin D deficiency.

Our study is unique in that it focuses exclusively on discordant imaging cases, a subgroup that is both challenging and clinically important. Although prior meta-analyses have shown the overall superiority of FCH PET/CT compared with MIBI, few investigations have specifically addressed discordant scenarios. Our results therefore add evidence that FCH PET/CT is particularly useful when conventional modalities are inconclusive. This study adds a distinct contribution to the current literature by focusing solely on discordant imaging cases, which have been largely overlooked in previous reports. By separating these cases from concordant ones, we were able to reveal practical differences in diagnostic behavior and surgical outcomes that are often hidden when the two are analyzed together (13, 14, 24).

These results are consistent with previous studies reporting the diagnostic superiority of FCH PET/CT (13, 15), particularly in patients with inconclusive or negative findings on conventional imaging (9, 15). FCH PET/CT offers several advantages, including higher spatial resolution, shorter imaging time, and a unique uptake mechanism driven by choline kinase activity, which is upregulated in hyperfunctioning parathyroid tissue (6). In contrast, MIBI SPECT/CT uptake is largely influenced by mitochondrial activity and content, leading to wide variability in sensitivity, especially in cases of multiglandular disease or low oxyphilic cell composition (6, 16–18). Our pathological analysis showed that the FCH PET/CT-concordant group (Group 3) had a higher proportion of chief cells (91.94% vs 82.65%) and a lower proportion of oxyphilic cells (9.03% vs 18.26%) compared with the double-concordant group (Group 1). Although these differences did not reach statistical significance (p=0.098), the trend suggests a possible association between cellular composition and imaging performance. Future studies with larger, matched cohorts will be needed to validate this observation. Chief cells are known to have higher metabolic activity and choline turnover than oxyphilic cells, resulting in increased FCH PET/CT uptake (6, 19). These histologic differences, while not statistically significant, may partially explain the improved concordance and diagnostic performance of FCH PET/CT in discordant cases. Further studies with larger, matched cohorts are needed to confirm this association.

Another important finding was the higher preoperative PTH levels observed in the FCH PET/CT-concordant group (Group 3) compared to the MIBI SPECT/CT-concordant group (Group 2). This difference may reflect more active hyperfunctioning parathyroid tissue, which in turn may facilitate FCH PET/CT uptake due to increased choline metabolism. The association between elevated PTH levels and FCH PET/CT positivity has been previously reported and supports the notion that FCH PET/CT is particularly effective in metabolically active disease (20). These findings suggest that the superior performance of FCH PET/CT is not solely attributable to its imaging resolution or technical capabilities, but also in its ability to reflect the physiological activity of hyperfunctioning parathyroid tissue (21). From a clinical perspective, these findings underscore the value of FCH PET/CT as a localization tool, especially in patients with discordant or inconclusive findings on conventional imaging (22). Accurate localization is critical for the success of minimally invasive parathyroidectomy and for reducing operative time and morbidity. Our results suggest that FCH PET/CT may improve surgical outcomes by enhancing preoperative confidence in localization, thereby increasing cure rates (23, 24). In our cohort, ultrasonography demonstrated concordance with surgical findings in approximately half of the patients. Importantly, most discordant cases were attributable to the absence of a detectable parathyroid lesion on ultrasound. This observation highlights the inherent limitation of ultrasonography in detecting small or ectopic hyperfunctioning glands and supports the complementary role of FCH PET/CT when conventional modalities fail to identify a candidate lesion.

Although our study focused on discordant cases where FCH PET/CT showed advantages, it is important to note that MIBI SPECT/CT remains a well-established and widely used modality. In concordant or straightforward cases, MIBI continues to demonstrate high sensitivity and specificity, and it provides reliable localization in many clinical settings. In addition, MIBI is generally less expensive and more widely available than FCH PET/CT, which makes it an accessible first-line option in most institutions. Our results should therefore be interpreted as complementary to, rather than replacing, the established role of MIBI SPECT/CT in routine practice. However, the higher upfront cost of FCH PET/CT may be offset by its superior localization accuracy in discordant cases, potentially reducing operative time, need for re-exploration, and overall healthcare utilization. Previous cost-effectiveness analyses have suggested that improved preoperative localization can lead to indirect savings through shorter hospital stays and fewer additional imaging studies. As the availability of FCH PET/CT increases, its relative cost is expected to decrease, further supporting its practical adoption in selected patients (25).

Despite these promising results, several limitations should be considered. First, the study was retrospective in nature and conducted at a single institution, which may introduce selection bias and may limit the generalizability of our findings. Second, although intraoperative findings and pathology were used as the reference standard, surgical decisions may also have been influenced by preoperative imaging and adjunctive tests such as ultrasonography, 4D CT, parathyroid venous sampling, or intraoperative PTH monitoring. These additional studies may have introduced incorporation bias, making it harder to assess the diagnostic performance of FCH PET/CT versus MIBI SPECT/CT in isolation. This type of incorporation bias occurs when imaging results influence the reference diagnosis, potentially overestimating the true diagnostic accuracy of the modality. Because surgeons were aware of preoperative imaging findings, FCH PET/CT results might have guided intraoperative exploration and lesion identification. Although pathology confirmation was used as the final standard to minimize bias, a completely blinded design would further strengthen the validity of future research. Third, intraoperative PTH kinetics and postoperative PTH and calcium at 6 and 12 months were used as surrogate markers for cure. Although this provides a broader evaluation, it may still miss persistence or recurrence, and misclassification is possible, as elevated PTH with normal calcium can reflect secondary causes. Longer-term prospective follow-up will still be required to confirm the durability of cure and to detect late recurrence.

Fourth, the number of patients in each group was uneven. In particular, Group 2 included only 5 patients, making statistical comparison with Group 3 underpowered; therefore, the observed difference in cure rates (80% vs. 95%) should be interpreted with caution. Future studies with larger cohorts may allow for matched comparisons between groups, which could yield more robust and meaningful insights. More broadly, the relatively small size of some subgroups means that many of our comparisons were likely underpowered. Fifth, surgical outcomes were assessed up to 12 months using both PTH and calcium levels. While this provides a more comprehensive evaluation than short-term follow-up alone, it may still not fully capture late recurrence or persistence. Longer-term prospective studies will be needed to confirm the durability of cure and to detect delayed disease recurrence. Sixth, although this study focused on the comparison between MIBI SPECT/CT and FCH PET/CT, the potential contribution of other localization methods, such as neck ultrasonography, was not incorporated into the analysis. The absence of such data may introduce bias, and future studies should consider integrating multimodal imaging results for a more comprehensive evaluation. Seventh, interobserver variability in image interpretation was not formally assessed, which may limit the reproducibility of our findings. Lastly, the cost-effectiveness of MIBI SPECT/CT and FCH PET/CT was not evaluated, which may be an important factor for clinical decision-making and broader implementation (25). In our institution, the cost of FCH PET/CT is approximately 562 USD with insurance and 985 USD without, compared with 306 USD and 661 USD for MIBI SPECT/CT, resulting in a difference of about 250–300 USD. Wider adoption is expected to reduce its cost over time, potentially improving cost-effectiveness. Further studies should evaluate the economic impact of incorporating FCH PET/CT into localization strategies.

## Conclusion

In patients with primary hyperparathyroidism, FCH PET/CT demonstrated superior diagnostic performance and higher postoperative cure rates compared to MIBI SPECT/CT in cases with discordant imaging findings. This advantage may be attributed not only to its imaging resolution, but also to its potential to capture physiological and pathological features that reflect glandular activity. While FCH PET/CT is not definitively superior in all clinical situations, it appears to provide greater confidence in preoperative localization, particularly when conventional imaging yields discordant results. Given these findings, it seems reasonable to consider FCH PET/CT as a preferred option in selected patients, although additional studies with larger cohorts and longer follow-up are needed to clarify its role in complex diagnostic settings.

## Data Availability

The original contributions presented in the study are included in the article/**Supplementary Material**. Further inquiries can be directed to the corresponding author.
